# Omadacycline Is Highly Active *In Vitro* against Mycoplasma genitalium

**DOI:** 10.1128/spectrum.03654-22

**Published:** 2022-10-31

**Authors:** Ken B. Waites, Donna M. Crabb, T. Prescott Atkinson, William M. Geisler, Li Xiao

**Affiliations:** a Department of Pathology, University of Alabama at Birmingham, Birmingham, Alabama, USA; b Department of Pediatrics, University of Alabama at Birmingham, Birmingham, Alabama, USA; c Department of Medicine, University of Alabama at Birmingham, Birmingham, Alabama, USA; INTHERES

**Keywords:** *Mycoplasma genitalium*, omadacycline, antimicrobial resistance, tetracyclines, macrolide, quinolone, antimicrobial treatment, cervicitis, pelvic inflammatory disease, urethritis

## Abstract

Here, we performed *in vitro* susceptibility testing on 10 Mycoplasma genitalium isolates against omadacycline, minocycline, tetracycline, doxycycline, moxifloxacin, levofloxacin, and azithromycin. Omadacycline was the most potent agent, with all MICs of ≤0.5 μg/mL. MICs were not affected by resistance to other agents, including resistance to other tetracycline class drugs. Omadacycline may be a potential treatment option for M. genitalium infection.

**IMPORTANCE** There are very few clinical isolates of Mycoplasma genitalium available for *in vitro* susceptibility testing. We studied 10 isolates and determined that the new semisynthetic aminomethylcycline omadacycline is active against isolates that are resistant to tetracyclines, macrolides, and quinolones. These data suggest that clinical studies should be performed in order to see if omadacycline may be useful to treat urogenital infections caused by M. genitalium.

## OBSERVATION

Mycoplasma genitalium causes urethritis and cervicitis and has been associated with reproductive sequelae (1). CDC-recommended treatments for M. genitalium include doxycycline, azithromycin, and/or moxifloxacin, depending on whether resistance has been documented and the clinical syndrome being treated (2). Increasing resistance to macrolides and fluoroquinolones and poor clinical efficacy with doxycycline underscore the urgent need for new treatments (3).

Omadacycline is a semisynthetic aminomethylcycline antibiotic that is active against a broad spectrum of Gram-positive and Gram-negative bacteria and some anaerobes, as well as atypical bacteria, including *Legionella*, Chlamydia, Mycoplasma pneumoniae, Mycoplasma hominis, and *Ureaplasma* species (4–8). The C-7 modification of the tetracycline D-ring circumvents the tetracycline efflux pump resistance mechanism (e.g., *tetK*), and the C-9 modification circumvents the ribosomal protection resistance mechanism (e.g., *tetM*) (9). Omadacycline is FDA cleared and indicated for community-acquired bacterial pneumonia and acute bacterial skin and soft tissue infection. The proportion of administered omadacycline dose excreted in the urine, its extensive tissue distribution, a cystitis study showing favorable outcomes with omadacycline treatment, and *in vitro* activity against other mycoplasmal species (6, 10) all suggest omadacycline may be beneficial in treating M. genitalium infections. Moreover, omadacycline is a derivative of minocycline, and minocycline has been shown to effectively treat some multidrug resistant M. genitalium infections (11). Our study determined MICs for omadacycline and other agents against M. genitalium reference strains and clinical isolates, including multidrug-resistant strains.

Antimicrobials were obtained in powdered form of known purity and dissolved per the manufacturer’s instructions. Stock solutions were prepared and used to make dilutions in 96-well microtiter plates. Agents tested included omadacycline, minocycline, tetracycline, doxycycline, moxifloxacin, levofloxacin, and azithromycin. Inoculum preparation, broth microdilution MIC assays, and quality-control procedures were performed by methods established for human mycoplasmas by the Clinical and Laboratory Standards Institute (CLSI) guideline M43-A (12). Some modifications were necessary due to the very slow growth of M. genitalium. All organisms were stored frozen at −80°C until thawed to room temperature for testing. M. genitalium ATCC reference strain 33530 (G37) was used for quality control. Additional quality-control MICs were determined using *M. hominis* ATCC 23114, which has reproducible MICs and designated MIC reference ranges for various drugs. Clinical isolates of M. genitalium are extremely rare due to difficulty in isolating them in culture from clinical specimens. Therefore, reference strains (*n* = 5), older stored clinical isolates (*n* = 2), and recent clinical isolates from patients undergoing testing at the University of Alabama at Birmingham (UAB) Diagnostic Mycoplasma Laboratory (*n* = 4) were used. There were three multidrug-resistant isolates obtained at different time points from an immunocompromised male who had failed multiple treatments that included doxycycline, azithromycin, and moxifloxacin and an additional multidrug-resistant isolate from another immunocompromised male who had received doxycycline. For these four drug-resistant M. genitalium isolates, mutations in 23S rRNA associated with macrolide resistance and mutations in the quinolone resistance-determining regions of *gyrA*, *gyrB*, *parC*, and *parE* were determined by Sanger sequencing of the corresponding genes (13). The 16S rRNA gene sequence was also tested for mutations (14), and the presence of the *tetM* element was assessed by PCR (15).

MIC data are shown individually in Table 1 and are summarized in Table 2. Omadacycline was potent against all M. genitalium strains, with MICs of ≤0.5 μg/mL (Table 2). Four M. genitalium isolates, of which three were recovered from a single patient at different time points, had mutations conferring resistance to azithromycin (MICs, 2 to 16 μg/mL), levofloxacin (MICs, 4 to 8 μg/mL), and moxifloxacin (MICs, 2 to 4 μg/mL). Tetracycline MICs (8 to 16 μg/mL) for these isolates were also 2- to 4-fold higher than the highest MIC for the isolates that were susceptible to macrolides and fluoroquinolones (4 μg/mL). Omadacycline MICs (0.063 to 0.125 μg/mL) were unaffected by resistance phenotype. Minocycline MICs (0.25 μg/mL) for two of the four multidrug-resistant isolates were similar to those obtained for the other six isolates, but the other two multidrug-resistant isolates had minocycline MICs of 0.5 to 1 μg/mL, which were 2- to 4-fold higher than the highest minocycline MIC for the isolates that were susceptible to macrolides and fluoroquinolones (0.25 μg/mL). Doxycycline MICs were 0.125 to 0.25 μg/mL for isolates for which tetracycline MICs were ≤4 μg/mL and were 2- to 8-fold higher (0.5 to 2 μg/mL) in four isolates that exhibited very high MICs for tetracycline (8 to 16 μg/mL).

**TABLE 1:** Individual MIC data for omadacycline and comparators against Mycoplasma genitalium^*a*^

Strain	Yr	Body site	MICs (μg/mL) of:							Genetic alterations^*b*^ of:					
			Oma	Min	Tet	Dox	Azi	Lev	Mox	16S rRNA	23S rRNA	*gyrA*	*gyrB*	*parC*	*parE*
ATCC 33530-G37 control	1980	Urethra	0.125	0.125	0.5	0.125	≤0.004	1	0.125						
JB	1980	Urethra	0.5	0.25	0.5	0.125	≤0.004	1	0.063						
ATCC 19896-TW10	1974–1975	Throat	0.25	0.25	1	0.25	≤0.004	2	0.125						
ATCC 49897-R32G	1974–1975	Throat	0.25	0.125	0.5	0.125	≤0.004	2	0.063						
ATCC 49898-TW48-5G	1974–1975	Throat	0.125	0.25	0.5	0.25	≤0.004	1	0.125						
ATCC 49895-M30	1980	Urethra	0.063	0.063	0.5	0.125	≤0.004	2	0.063						
M2341	1991	Urethra	0.125	0.25	4	0.25	≤0.004	0.25	0.032						
UAB-73697^*c*^	2018	Urine	0.125	1	8	0.5	4	8	2	C1440T	A2072G	WT	C1384T (P462S)	G248T (S83I)	WT
UAB-75956^*c*^	2019	Urine	0.125	0.5	16	2	16	8	4	C1440T	A2072G	WT	C1384T (P462S)	G248T (S83I)	WT
UAB-84535^*c*^	2021	Urine	0.063	0.25	16	1	2	4	2	C1440T	A2072G	WT	C1384T (P462S)	G248T (S83I)	WT
UAB-84211	2020	Urine	0.063	0.25	8	0.5	4	8	4	WT	A2071G	G295T (D99Y)	A1483G (I495V)	G248T (S83I)	WT

a *n* = 10; Oma, omadacycline; Min, minocycline; Dox, doxycycline; Azi, azithromycin; Lev, levofloxacin; Mox, moxifloxacin; WT, wild type, no mutations; ATCC, American Type Culture Collection.

b Sequences were compared to strain ATCC 33530-G37.

c Isolates obtained at different time points from the same patient.

**TABLE 2:** MIC summary data for antimicrobial agents tested against Mycoplasma genitalium

Parameter	MIC (μg/mL) of:						
	Omadacycline	Minocycline	Tetracycline	Doxycycline	Azithromycin	Levofloxacin	Moxifloxacin
Range	0.063 to 0.5	0.063 to 1	0.5 to 16	0.125 to 2	≤0.004 to 16	0.25 to 8	0.032 to 4
MIC_50_	0.125	0.25	1	0.25	≤0.004	2	0.125
MIC_90_	0.25	0.5	16	1	4	8	4

Our study is the first to our knowledge to determine MICs of omadacycline for M. genitalium. We found that omadacycline had potent *in vitro* activity against M. genitalium, with very low MIC values against all strains tested, including multidrug-resistant strains for which omadacycline MICs were severalfold lower than other drugs in the tetracycline class. Most clinical experience and research to date using a tetracycline class antibiotic for treatment of M. genitalium have been with doxycycline, which has been found to have poor clinical and microbiological efficacy despite relatively low MICs (3, 16). However, limited case reports show minocycline can sometimes be an effective M. genitalium treatment in the setting of doxycycline failure (11, 17). Minocycline is also known to be more efficacious than doxycycline against some other bacteria, such as methicillin-resistant Staphylococcus aureus and Acinetobacter, despite having similar MIC values (18). Differences in antibacterial efficacy between minocycline and doxycycline could possibly relate to differing pharmacokinetic/pharmacodynamic parameters and tissue penetration.

Based on our clinical experience, there are also patients with M. genitalium infection who experience treatment failure with minocycline; it is possible in some that the M. genitalium strains may be tetracycline-resistant like the strains we tested here (Table 1). However, in most cases of M. genitalium treatment failure, it is unknown whether the strain is tetracycline resistant because the strain cannot be grown in culture for susceptibility testing, and there is no genotypic marker currently available for detecting a tetracycline-resistant strain. We did not detect the *tetM* sequence in the tetracycline-resistant isolates we studied, and we are currently investigating other tetracycline resistance mechanisms to determine a genotypic marker(s) for tetracycline resistance. Our data suggest that omadacycline may be a potential treatment alternative for infections with multidrug-resistant M. genitalium infections that do not respond to other agents, and our results justify clinical studies to investigate this possibility.
